# Comparison of Fabrication Methods of Metal-Organic Framework Optical Thin Films

**DOI:** 10.3390/nano8090676

**Published:** 2018-08-30

**Authors:** Yan Huang, Cheng-an Tao, Rui Chen, Liping Sheng, Jianfang Wang

**Affiliations:** 1 National & Local Joint Engineering Laboratory for New Petro-chemical Materials and Fine Utilization of Resources, College of Chemistry and Chemical Engineering, Hunan Normal University, Changsha 410081, China; 15211097117@163.com; 2 College of Liberal Arts and Science, National University of Defense Technology, Changsha 410073, China; chenrui95@live.com

**Keywords:** metal-organic frameworks, thin film, optical property, fabrication method

## Abstract

Homogeneous metal-organic frameworks (MOFs)-based optical thin films have attracted increasing attention, since they can potentially be used as active components in optical/opt-electrical devices, and how to fabricate MOF thin films with high quality is the premise of practically using them. Herein, five fabrication methods of MOF films are systematically investigated and compared from the aspects of appearance, reflectivity, micro-morphology, surface roughness, and optical properties of the films. The famous robust Zr-based MOF, UiO-66 (UiO = University of Oslo) is chosen as a model, and the five methods are spin-coating, dip-coating, self-assembly, direct growth, and the stepwise layer by layer growth method. This study provides fundamental support for the application of MOFs in the optical field.

## 1. Introduction

Metal-organic frameworks (MOFs) are a class of inorganic-organic hybrid crystalline materials that consist of metal ions or clusters and organic bridging ligands via coordinate-covalent bonds [1,2]. They have exhibited great potential for applications in many fields, such as gas storage [3], catalysis [4,5], drug delivery [6], chemical sensing [7,8,9,10], and so on, benefitting from their outstanding features, including ultrahigh porosity, large internal surface area, good thermal stability, and high chemical tailorability [11,12,13].

**Scheme 1:**
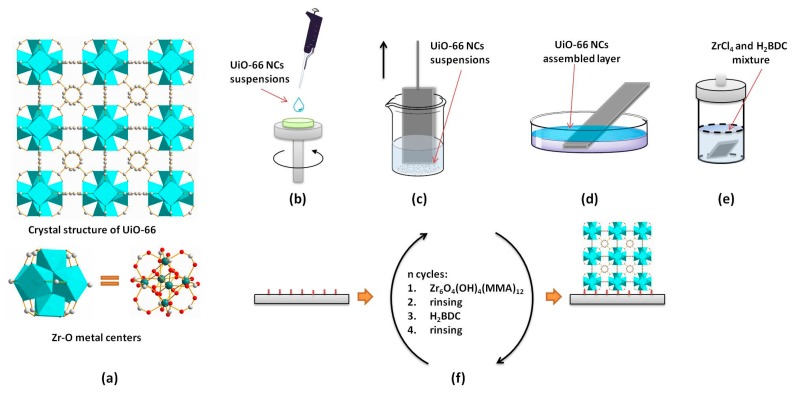
(**a**) Crystal structure of UiO-66, consisting of Zr-O metal centers connected by terephthalate linkers. Zr, cyan; C, gray; O, red; H, omitted. (**b**–**f**) Illustration of five fabrication methods of metal-organic framework (MOF) optical thin film: (**b**) spin-coating, (**c**) drop-coating, (**d**) self-assembly, (**e**) direct growth, (**f**) stepwise layer by layer growth method.

Optical thin films (OTFs) are composed of single or multi-layered media with uniform thicknesses [14]. They form the basis of developing a variety of optical elements and devices, such as anti-reflection coatings, high reflectivity films, polarizing films, long/short-pass filters, and beam splitters [15]. The countless types, the tunable optical properties, and the above-mentioned unique characteristics of MOFs make them a promising new generation of optical thin film materials. But, how to fabricate MOF optical thin films with high quality (low roughness) is a chief and fundamental issue. Recently, homogeneous MOF-based thin films [16,17], particularly optical thin films [18,19,20,21,22,23], have attracted more and more attention, since they can potentially be used as active components (which can be easily regulated) in functional devices [16,17]. To date, several strategies have been developed for preparing MOF thin films. Férey and coworkers [18] first prepared flexible porous iron(III) trans, trans-muconate MIL-89 (MIL = Materials of Institut Lavoisier) colloidal optical thin films using the dip-coating method, and expanded on chromium terephthalate MIL-101(Cr) [19], chromium trimesate MIL-100(Cr) [20], and iron(III) trimesate MIL-100(Fe) [20]. Lotsch’s group [24] fabricated MOF thin films by spin-coating a MOF nanoparticle suspension onto a flat substrate. Two kinds of MOFs have been explored. One is copper trimesate (Cu_3_(BTC)_2_, also known as HKUST-1, HKUST = Hong Kong University of Science & Technology), which contains Cu(II)-paddlewheel-type nodes and trimesate struts, and the other one is the isoreticular metal-organic framework-3 (IRMOF-3, zinc amino-terephthalate). Our group [10,22,23,25] successfully applied this method to construct many MOF thin films, such as iron(III) terephthalate MIL-88B, aluminium terephthalate MIL-53, MIL-101(Cr), and their analogues. Huo et al. [9] developed a self-assembly method by dispersing zirconium(IV) terephthalate metal organic framework UiO-66 (UiO = University of Oslo) nanoparticles on the surface of water and transferring the self-assembled MOF thin film to a substrate. Lu et al. [8] constructed zeolitic imidazolate framework (ZIF)-8-based optical thin films via a direct growth method that functioned as selective sensors for chemical vapors and gases. Redel et al. [21] presented the optical properties of HKUST-1 thin film (also called surface-anchored metal-organic frameworks, SurMOF) which were fabricated by stepwise layer by layer growth onto a substrate. Although there have been a few successful cases to prepare MOF thin films, there is still a lack of systematic exploration on the performance and potential application of the thin films from the perspective of optical films. Moreover, for the same MOF system, there is a lack of detailed comparison on these preparation methods. In addition, the stability of optical thin films is vital for practical application. Generally, the MOFs have poor water stability, which hinders MOF thin film use in actual devices. A famous robust Zr-based MOF, UiO-66, based on terephthalate linkers and 12-coordinate cationic Zr_6_O_4_(OH)_4_^12+^ clusters (Scheme 1a) is optically transparent in the visible region and has exceptional mechanical, chemical, and thermal stabilities [13,26], especially aqueous stability over a range of pH values. If UiO-66 can be successfully prepared into a high quality optical film, it will be possible to apply it to actual optical or optoelectronic devices in the future.

In this study, UiO-66 was chosen as a model. The UiO-66 thin films were fabricated by five methods (Scheme 1b–f), i.e. spin-coating (SP), dip-coating (DP), self-assembly (SA), direct growth (DG), and the stepwise layer by layer growth (LBL) method, and the according thin films were denoted as OTF-SP, OTF-DP, OTF-SA, OTF-DG, and OTF-LBL, respectively. These optical thin films were compared from the aspects of appearance, reflectivity, micro-morphology, surface roughness, and optical properties. The OTF-SP film has a uniform color, greater reflectivity, flat microstructure, and minimal roughness. The quality of OTF-DP and OTF-SA films depends on the deposition times. The OTF-DG film has the highest roughness, which cannot meet the requirement for the use in optical films. The OTF-LBL film has the brightest color, a dense and smooth film surface, low surface roughness, and the maximum effective refractive index. This study provides fundamental support for the application of MOFs in the optical field.

## 2. Experimental Section

### 2.1. Materials

UiO-66 nanocrystals (NCs) were synthesized according to literature [12,27], and characterized by scanning electron microscopy (SEM), Fourier transform infrared (FTIR), X-ray diffraction (XRD), N_2_ adsorption-desorption isotherm and thermogravimetric analysis (Figure S1). The UiO-66 suspensions were prepared by dispersing the UiO-66 nanocrystals in EtOH. The 12-coordinate cationic Zr_6_O_4_(OH)_4_^12+^ clusters based precursor (Zr-precursor) [28,29] was prepared by reaction of methacrylic acid and Zr(PrO)_4_ and confirmed by XRD and FTIR. (Figures S6 and S7). All reagents were used as received without further purification.

Silicon wafers were cut into small pieces (~2 cm × 2 cm) which were used as substrates for fabrication of MOF thin films. The substrates were pre-cleaned with soap and water and subsequently treated with piranha solution (H_2_SO_4_/H_2_O_2_, volumetric ratio 7:3) at 70 °C for 1 h. After thorough rinsing with deionized water, the wafers were dried under air flow and stored in ethanol. For spin-coating, dip-coating, self-assembly deposition, and direct growth, the substrates were dried under nitrogen flow before use. For layer-by-layer growth, the substrates were functionalized with H_2_BDC by incubating in a *N*,*N*-dimethyl formamide (DMF) solution containing 33 mM H_2_BDC at room temperature for 3 h, and then cleaned with ethanol and dried with a stream of nitrogen gas before use.

### 2.2. Preparation of MOF Optical Thin Film

The OTF-SP films were fabricated by spin-coating 200 µL of UiO-66 nanocrystals alcoholic suspensions (4.3 wt. %) onto the substrate at 3000 rpm for 60 sec (Scheme 1b). The OTF-DP films were fabricated by dip-coating UiO-66 nanocrystals alcoholic suspensions (4.3 wt. %) using a withdrawal speed of one mm·s^−1^ (Scheme 1c). The deposition process was performed once, thrice, and five times, and the corresponding thin films were denoted as OTF-DP-1, OTF-DP-3, and OTF-DP-5, respectively. The OTF-SA films were prepared according to a previous report [9], in brief, the polyvinylpyrrolidone (PVP)-functionalized UiO-66 nanocrystals self-assembled on the water-air interface, and then the formed close-packed monolayer of UiO-66 nanocrystals was transferred onto the silicon substrate through the simple dip coating process (Scheme 1d). The process was repeated for one, two, or three times and the corresponding thin films were denoted as OTF-SA-1, OTF-SA-2, and OTF-SA-3, respectively. The OTF-DG films were hydrothermally prepared by putting the silicon substrate in the precursor solution (ZrCl_4_:terephthalic acid (H_2_BDC):H_2_O:acetic acid: DMF = 1:1:1:500:1500) (Scheme 1e). The OTF-LBL films were fabricated by dipping the functionalized substrate successively in (1) an ethanolic Zr-precursor (2 mM) solution, (2) absolute ethanol, (3) an ethanolic H_2_BDC (2 mM) solution, and again (4) absolute ethanol for 35 cycles (Scheme 1f). The experimental details were shown in Supplementary Materials.

### 2.3. Characterization

FTIR spectra were obtained using a Spectra Two spectrophotometer (PerkinElmer, Waltham, MA, USA) from 4000 cm^−1^ to 500 cm^−1^ with an attenuated total reflection (ATR) accessory. The XRD patterns were collected using a Ttr III type X-ray diffractometer (Rigaku, Tokyo, Japan) in θ–θ geometry with a graphite-monochromated CuK_α_ radiation source. The N_2_ adsorption-desorption isotherm of the samples at liquid nitrogen temperature (78 K) and gas saturation vapor tension range was measured by a BEL Mini sorption instrument (Bel Japan Inc., Osaka, Japan). TGA was completed by a STA6000 thermogravimetric analyzer (Perkin Elmer, Waltham, MA, USA) at a scanning rate of 10 °C min^−1^ under N_2_ atmosphere from 30 to 700 °C. A fiber spectrometer (USB2000+, Ocean optics, El Dorado Hills, CA, USA) coupled to an optical microscope (the numerical aperture of the 100X microscope objective is 0.80) was used to measure the specular reflectance in the 400–900 nm range at the normal incidence. SEM images were obtained using an S-4800 electron microscope (Hitachi, Ibaraki, Japan). The samples were sputtered with a thin layer of Au prior to imaging. The surface profiles were characterized using a profilometer (Talysurf PGI 1240, Taylor-Hobson, Leicester, UK) with a diamond stylus. Ellipsometry measurements performed with a XLS-100 ellipsometer (Woollam, Lincoln, NE, USA) at an angle of 75°, and within a spectral range of 400–900 nm at room temperature.

## 3. Results and Discussion

### 3.1. Appearance and Reflectivity

**Figure 1:**
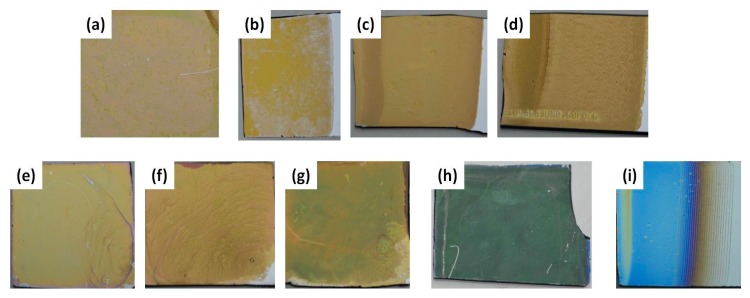
Photographs of MOF optical thin films. (**a**) OTF-SP, (**b**) OTF-DP-1, (**c**) OTF-DP-3, (**d**) OTF-DP-5, (**e**) OTF-SA-1, (**f**) OTF-SA-2, (**g**) OTF-SA-3, (**h**) OTF-DG, (**i**) OTF-LBL.

As shown in Scheme 1a, in perfect crystal structure of UiO-66, each Zr-O metal center [Zr_6_O_4_(OH)_4_ octahedron] is fully coordinated by 12 terephthalate linkers to form a highly connected framework. The UiO-66 nanocrystals exhibit octahedron morphology, that has a side length of approximately 340 nm (Figure S1). Compared with FT-IR spectra of H_2_BDC (Figure S2), the bands of protonated carboxylic groups at around 1,710 cm^−1^ disappear in UiO-66, indicating that the carboxyl groups of ligands have been coordinated with Zr-O metal center. The observed powder XRD pattern of UiO-66 (Figure S3) is consistent with the simulated pattern of the UiO-66 single crystal structure, which confirms the formation of pure MOFs. The isotherm of UiO-66 (Figure S4a) shows typical absorption of a microporous material. The calculated Brunauer–Emmett–Teller (BET) surface area of the UiO-66 nanocrystals is 1030.6 m^2^/g. Accordingly, the total pore volume was 0.445 cm^3^/g. Based on the desorption isotherms, the pore size of UiO-66 was 0.7 nm, as evaluated by the MP method (Figure S4b). UiO-66 nanocrystals also exhibits good thermal stability up to 400 °C (Figure S5). All these results confirm the success of the preparation of UiO-66 nanocrystals. In general, octahedron-shaped nanoparticles are more difficult to form homogenous and flat films than spherical nanoparticles. In previous reports on chemical solution deposition of MOF nanoparticles, most of the MOF particles used were ball shaped (or quasi-spherical) [10,18,19,20,22,23], such as MIL-89, MIL-100, MIL-53, MIL-101, HKUST-1, IRMOF-3 etc. Here, octahedral UiO-66 nanocrystal-based thin films were fabricated by spin-coating, dip-coating, and the self-assembly method. Figure 1a shows the optical thin film fabricated by spin-coating, which displays a uniform pink color. The corresponding reflection spectrum is shown in Figure S8, the maximum of reflection of 800 nm reaches 95%. All OTF-DP films (OTF-DP-1, OTF-DP-3, and OTF-DP-5) show yellow (Figure 1b–d), but the coverage and surface morphology are different. For OTF-DP-1, the coverage is lowest (about 44%), which induces some areas of the substrate to be exposed. For OTF-DP-3, the surface is continuous and uniform. For OTF-DP-5, there are many pinholes on the surface, and is a darker yellow color. The reflectance spectra of them are very similar (Figure S9), which agrees with their colors. The OTF-SA shows varied color for different deposition times, from light yellow (once), dark yellow (twice), to yellowish green (trice) (Figure 1e–g). The corresponding reflection spectra have reflection peaks at 574 nm, 598 nm, and 567 nm, respectively (Figure S10). Besides, the MOF thin films were also constructed by hydrothermal direct growth, the obtained OTF-DG shows dark green and looks lackluster (Figure 1h), which is in agreement with its reflection spectrum which has no apparent reflections between 400 nm and 700 nm (Figure S11). The last method for preparing MOF thin films is stepwise layer-by-layer, and the produced thin film (OFT-LBL) shows vivid blue (Figure 1i), and there is a reflection peak at 450 nm (Figure S12). The reflection spectrum of OFT-LBL is the smoothest among these thin films. The smooth spectrum means there is less light scattering by the film because of the absent of obvious aggregations or cracks. This result is in agreement with SEM observation, further suggesting the OFT-LBL film is uniform and compact.

### 3.2. Surface Micro-Morphology

**Figure 2:**
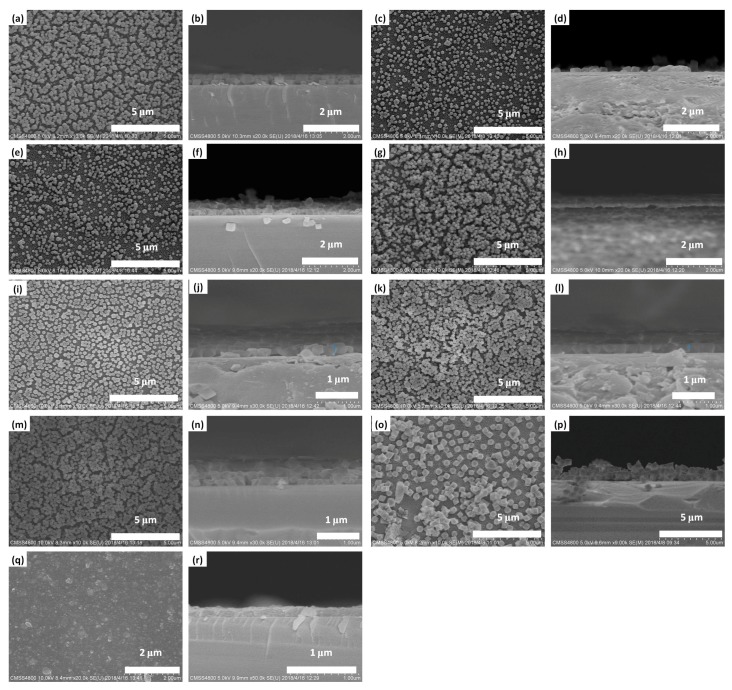
Top-view (**a**,**c**,**e**,**g**,**i**,**k**,**m**,**o**,**q**) and side-view (**b**,**d**,**f**,**h**,**j**,**l**,**n**,**p**,**r**) SEM images of MOF optical thin films. (**a**,**b**) OTF-SP, (**c**,**d**) OTF-DP-1, (**e**,**f**) OTF-DP-3, (**g**,**h**) OTF-DP-5, (**i**,**j**) OTF-SA-1, (**k**,**l**) OTF-SA-2, (**m**,**n**) OTF-SA-3, (**o**,**p**) OTF-DG, (**q**,**r**) OTF-LBL.

Surface micro-morphologies of the optical thin films were observed by SEM, as shown in Figure 2. From the top view of SEM images (Figure 2a,g,m), the surface micro-morphologies of OTF-SP, OTF-DP-5, and OTF-SA-3 are almost the same, and UiO-66 nanocrystals gather into small domains with obvious gaps between the domains. When the film is dried, UiO-66 colloidal particles are more likely to attract each other and accumulate due to the capillary force, and induced the cracks between the domains. As a whole, the film is still relatively flat. The flatness (to represent with standard deviation of the thickness of the film measured in the side-view SEM images) of OTF-SP (14 nm) is better than that of OTF-DP-5 (19 nm), which is better than that of OTF-SA-3 (21 nm). For the film deposited only once by dip-coating (Figure 2c), the surface coverage is only 44.4%, estimated from the black and white binary SEM images with Image J software [30]. The discrete UiO-66 nanocrystals are distributed across the surface of the substrate. For OTF-DP-3 film (Figure 2e), some areas show the accumulation of UiO-66 nanocrystals. Therefore, under our experimental conditions, more than five depositions are required to obtain a more uniform film. For the self-assembly method, a single-layer film of MOFs can be obtained by the self-assembly once (Figure 2i). When self-assembling twice, the nanocrystals of the second layer are randomly deposited into the gap between the first layer of nanocrystals to improve surface coverage of the film (Figure 2k). From the side view of the SEM image (Figure 2b), the film obtained by the spin coating process is basically composed of two layers of UiO-66 nanocrystals, with a thickness of about 380 ± 14 nm. The OTF-DP-1 film (Figure 2d) is primarily composed of sparse monolayers of nanocrystals and has a thickness of about 175 ± 47 nm. The OTF-DP-3 film (Figure 2f) is not uniform, with both monolayer and multi-layer stacking present. The OTF-DP-5 film (Figure 2h) is mostly composed of double-layered nanocrystals with a thickness of about 503 ± 19 nm. During self-assembly deposition, the number of nanocrystal layers of the film corresponds to the number of assemblies. The OTF-SA-1 is composed of a single layer of nanocrystals and has a thickness of about 252 ± 25 nm (Figure 2j). When it is assembled twice, it is mostly composed of interleaved double-layered nanocrystals with a thickness of about 498 ± 22 nm (Figure 2l). When assembled three times, the resulting film consists of three layers of nanocrystals and the thickness increased to about 606 ± 21 nm (Figure 2n). Unlike the solution-based deposition methods of MOF nanoparticles, the film obtained by the direct growth method consists of a single layer of UiO-66 crystals with varied sizes, as shown in Figure 2o. The large grains are about one micron, while the small grains are less than 200 nm. The surface is very rough, and the uneven thickness of the film can also be seen from the side-view SEM image (Figure 2p). The OFT-LBL film obtained after 35 cycles looks very dense (Figure 2q), there are no cracks or pinholes on the surface. Generally, the film is very uniform. From the side view (Figure 2r), it is also seen that the film is dense and identical with a thickness of about 153 ± 7 nm and a cycle of about 4.3 nm. From the above results, we can see that the three methods based on the deposition of nanoparticles can obtain relatively flat optical thin films, but the surface has cracks; and the film generated by direct growth has a large roughness (40 nm), low coverage (~50%), and uneven thickness, making it unable to meet the requirements of optical films; while the layer-by-layer deposited film is smooth, uniform, and has the best coverage and compactness.

### 3.3. Surface Roughness

Furthermore, the qualities of the films were quantitatively analyzed using surface roughness. The surface roughness was determined using a profilometer with a diamond stylus. The sampling length and evaluating length were 0.08 mm and 2 mm, respectively. The total evaluating length was 10 mm for evaluating five times. The roughness profiles of UiO-66-based thin films are shown in Figures S13–S21. The arithmetic average roughness, *R*_a_ is the arithmetic average value of filtered roughness profile and the root mean squared roughness, *R*_q_ is root mean squared value of filtered roughness profile. The calculated *R*_a_ and *R*_q_ are listed in Table 1. The OTF-SP film has the best flatness, while the OTF-DG has the worst roughness (*R*_a_ = 40.3 nm). The roughness of MOF thin films made of octahedral UiO-66 nanocrystals is generally higher than that of spherical MOF nanoparticles-based thin films [19,22,23]. For the dip-coated films, the more depositions, the larger the roughness due to the accumulation, which is consistent with the SEM observation. For the self-assembled films, the trend is just the opposite. Their roughness decreases with the increase of depositions, thanks to the interleaved filling of following nanocrystals, which is in agreement with the micro-morphology.

### 3.4. Optical Properties

**Figure 3:**
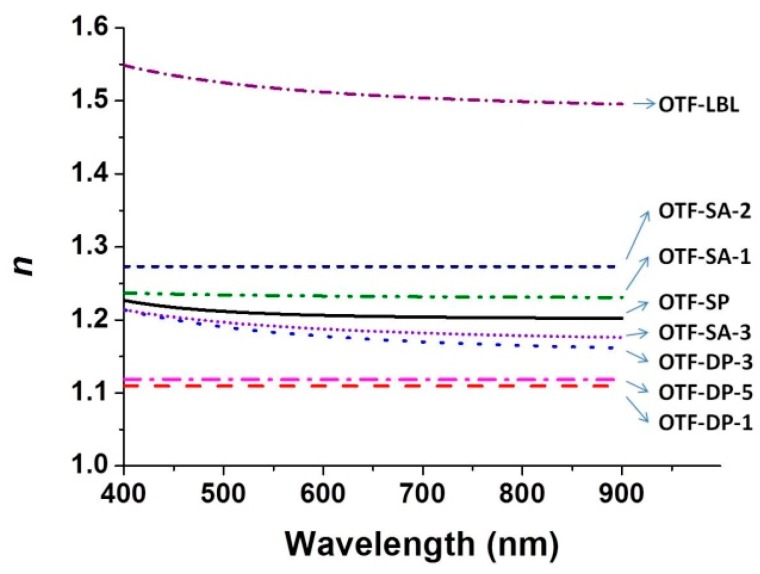
Efficient refractive index of MOF optical thin films.

The optical constants of the MOFs-based thin films were explored by spectroscopic ellipsometry. The ellipsometric parameters (*Psi*, *Delta*) were obtained in the wavelength range of 400–900 nm at an incident angle of 75°. A Cauchy model was chosen to fit the experimental data. The experimental and generated ellipsometric parameters of the films are presented in Figures S22–S26. The fitting of all MOF thin films present sufficient agreement with the experimental data over the entire measured spectral range, except the film produced by direct growth (Figure S26), suggesting that OTF-DG films cannot meet the requirement of the optical films due to their high roughness. The corresponding thicknesses evaluated via ellipsometry were 379 ± 5 nm (OTF-SP), 285 ± 9 nm (OTF-DP-1), 462 ± 7 nm (OTF-DP-3), 813 ± 4 nm (OTF-DP-5), 273 ± 1 nm (OTF-SA-1), 545 ± 5 nm (OTF-SA-2), 829 ± 6 nm (OTF-SA-3), and 153 ± 2 nm (OTF-LBL), respectively. The variation tendency of the thickness agrees well with the SEM observation. The roughness of the thin films led the ellipsometry method to overestimate their thicknesses because of the high domain on the surface of thin film would be recognized as the surface level. Interestingly, benefiting from the dense and smooth surface of the OTF-LBL film, the thicknesses of OTF-LBL film estimated from SEM observation and ellipsometry method are almost equal. The obtained optical constants for the MOF thin film as a function of wavelength are shown in Figure 3 and Figure S27. In general, the refractive index (*n*) of the films has this order: OTF-DP < OTF-DP < OTF-SA < OTF-LBL. The *n* of OTF-LBL has the highest value, which can be considered as the intrinsic refractive index of UiO-66 due to the compactness and integrity of the film. For convenience of discussion, the average index values (from 400 nm to 900 nm) and that at the wavelength of 900 nm (the longest wavelength in the measured range) for all samples are listed in Table 2. The nanocrystal-based films can achieve a refractive index lower than 1.23, which is important for application as antireflection film. Such low refractive index benefits from the cracks and voids between the UiO-66 nanocrystals. Assuming the intrinsic refractive index of UiO-66 was 1.5120, the index of air is 1, the void percent in the film was estimated according to the effective medium theory [13,23,31]. The results are shown in Table 2. We can see that the voids of OTF-SP, OTF-DP, and OTF-SA films are about 25%, 30%, and 23% respectively.

## 4. Conclusions

In summary, the UiO-66-based thin films have been successfully prepared using five different methods. The OTF-SP film has a uniform color, greater reflectivity, flat microstructure, and minimal roughness. The quality of the OTF-DP film is related to the number of depositions. When the deposition times are few, the surface coverage is low, and when increasing the times of deposition, a more homogenous film can be obtained. The OTF-SA film has a uniform color, and the number of MOF layers is in direct proportion to the times of assembly, while the roughness decreases as the number of assembly increases, thanks to the interleaved filling of following nanocrystals. All the above three methods based on solution deposition of MOF nanoparticles have made a thin film with a lower refractive index (even < 1.22) and have the potential for application in antireflection films and other devices. The surface of the OTF-DG film is dark in color and has the highest roughness, which does not meet the requirements for use of optical films. The OTF-LBL has the brightest color, a flowing reflection spectrum curve (small scattering), dense and smooth film surface, low surface roughness, maximum effective refractive index, but small thickness, and a growth efficiency of about 3.4 nm per growth cycle.

## Figures and Tables

**Table 1:** Roughness of MOF optical thin films.

Samples	*R*_a_(nm)	*R*_q_(nm)
OTF-SP	8.7 ± 1.3	12.1 ± 1.9
OTF-DP-1	7.6 ± 0.7	9.7 ± 1.0
OTF-DP-3	13.8 ± 5.1	17.7 ± 6.4
OTF-DP-5	25.5 ± 4.8	42.2 ± 16.8
OTF-SA-1	15.6 ± 2.0	19.5 ± 2.3
OTF-SA-2	15.2 ± 1.9	21.5 ± 0.7
OTF-SA-3	10.9 ± 2.5	19.2 ± 9.8
OTF-DG	40.3 ± 6.2	56.3 ± 9.6
OTF-LBL	14.2 ± 1.4	19.3 ± 7.9

**Table 2:** Average efficient refractive index (*n*_ave_), specific efficient refractive index at 900 nm (*n*_900_), and calculated voids of MOF optical thin films.

Samples	*n*_ave_	*n*_900_	*V*_void_ (%)
OTF-SP	1.208 ± 0.024	1.2022	25.19
OTF-DP-1	1.110 ± 0.016	1.1095	32.11
OTF-DP-3	1.178 ± 0.012	1.1616	27.35
OTF-DP-5	1.118 ± 0.015	1.1182	31.51
OTF-SA-1	1.233 ± 0.009	1.2308	23.32
OTF-SA-2	1.273 ± 0.015	1.2731	20.25
OTF-SA-3	1.188 ± 0.012	1.1760	26.64
OTF-LBL	1.512 ± 0.053	1.4955	0

## Supplementary Materials

The following are available online at http://www.mdpi.com/2079-4991/8/9/676/s1, experimental details, characterization of UiO-66 and Zr-precursor, reflection spectra, SEM images and generated and fitted curves of ellipsometry.
