# Supplementary material for: Comparison of Fabrication Methods of Metal-Organic Framework Optical Thin Films
Source: Nanomaterials (Basel). 2018 Aug 30;8(9):676. doi: 10.3390/nano8090676 (PMC6164412; doi:10.3390/nano8090676)
Supplement: Supplementary file 1 [file nanomaterials-08-00676-s001.pdf]

Electronic Supplementary Information (ESI)

Comparison of Fabrication Methods of Metal-Organic Framework Optical Thin Films

Yan Huang [1,†], Cheng-an Tao [2,*,†], Rui Chen [2], Liping Sheng [1,*] and Jianfang Wang [2,*]

[1] National & Local Joint Engineering Laboratory for New Petro-chemical Materials and Fine Utilization of Resources, College of Chemistry and Chemical Engineering, Hunan Normal University, Changsha 410081, China; 15211097117@163.com

[2] College of Liberal Arts and Sciences, National University of Defense Technology, Changsha 410073, China; chenrui95@live.com

* Correspondence: tca02@mails.thu.edu.cn (C.T.); sleeping1217@126.com (L.S.); wangjianfang@nudt.edu.cn (J.W.); Tel.: +86-731-8457-4241 (J.W.)

† These authors contribute to this work equally.

1 Experimental Section

1.1 Materials and methods

Zirconium chloride ($ZrCl_4$, 97%) was purchased from Acros. Zirconium propoxide ($Zr(PrO)_4$, 70% solution in n-propanol) was from Sigma-Aldrich. Methacrylic acid (MAA, 99%) was from Adamas-beta. Terephthalic acid (H_2BDC, 99%) was from Aladdin chemical. Triethylamine (TEA, AR), polyvinylpyrrolidone (PVP, Mw~55000, GR), acetic acid (≥99.5%), sulfuric acid (H_2SO_4, 98%), hydrogen peroxide (H_2O_2, ≥30%) and methanol were from Sinopharm. Sodium dodecyl sulfate (SDS, CP) was purchased from Xilong chemical. N,N-dimethylformamide (DMF, ≥99.5%) was from Hengxin chemical. Absolute ethanol (≥99.7%) was from Tongyong chemical. Deionized water was home-made. All reagents were used as received without further purification. Deionized water was used throughout the work.

1.2 Synthesis of UiO-66 nanocrystals

The synthesis of UiO-66 nanocrystals was performed according to literature[1, 2]. 49.8 mg of H_2BDC and 30 μL of TEA were dissolved in 5 mL of DMF while 66.8 mg of $ZrCl_4$ and 1.38 mL of acetic acid were dissolved in 5 mL of DMF separately. The solutions of H_2BDC and $ZrCl_4$ were combined in 20 mL vial, capped and placed in 85 ℃ oven for a day. The resultant UiO-66 nanocrystals were washed three times with DMF using a centrifuge (4,400 rpm for 20 min) and sonication, and then sequentially immersed in methanol for three 24 hrs periods. Finally, UiO-66 nanocrystals were activated by removing the solvent under vacuum for 12 hrs at room temperature, and the resultant products were as raw materials for subsequent MOFs film preparation by spin coating, dip-coating and self-assembly.

1.3 Synthesis of Zr-precursor

Synthesis of Zr-precursor, $Zr_6O_4(OH)_4(MAA)_{12}$ was performed according to literature[3, 4]. MMA (1.4 mL) was added to 2 mL $Zr(PrO)_4$ solution, with addition of a drop of water. Reaction mixture was stirred for 10 minutes using a magnetic stirrer in open flask and left to stand for one hour before the volume of the solution was reduced to 1/4 of a starting volume. The formed colorless solid was filtered under vacuum and washed once with 2 mL of *i*-propanol. Products, colorless crystalline materials were subjected to PXRD and FTIR-ATR analyses prior to further stepwise layer-by-layer growth.

1.4 Pre-treatment of substrates

Silicon wafers were cut into small pieces (~2 cm × 2 cm) which were used as substrates for fabrication of MOF thin films. The substrates were pre-cleaned with

soap and water and subsequently treated with piranha solution (H_2SO_4/H_2O_2, volumetric ratio 7:3) at 70 ℃ for one hour. After thoroughly rinsing with deionized water, the wafers were dried under air flow and stored in ethanol. For spin-coating, dip-coating, self-assembly deposition and direct growth, the substrates were dried under nitrogen flow before use. For layer-by-layer growth, the substrates were functionalized with H_2BDC by incubating in a DMF solution containing 33 mM H_2BDC at room temperature for 3 hours, and then cleaned with ethanol and dried with a stream of nitrogen gas before use.

1.5 Fabrication of Metal Organic Framework Thin Film

1.5.1 Spin-coating method

The MOF optical thin films were fabricated by spin-coating 200 μL of UiO-66 nanocrystals alcoholic suspensions with MOFs content of 4.3 wt.% onto the substrate at 3000 rpm for 60 s. The films were annealed at 200 ℃ for 20 min after coating, and denoted as OTF-SP.

1.5.2 Dip-coating method

The MOF optical thin films were fabricated by dip-coating UiO-66 nanocrystals alcoholic suspensions (4.3 wt%) at room temperature and under ambient atmospheric conditions, using withdrawal speed of 1 mm s^{-1}. The films were annealed at 200 ℃ for 20 min after coating. The deposition process was performed once, trice and five times, and the corresponding thin films were denoted as OTF-DP-1, OTF-DP-3 and OTF-DP-5, respectively.

1.5.3 Self-assembly method

Before assembly, the surface of UiO-66 nanocrystals were functionalized with PVP according to literature and redispersed in water/ethanol (v:v=1:1) solution. The self-assembly was carried out on water-air interface according to a previously reported procedure[5]. Briefly, an 8 mm×35 mm glass slide was leaned against the rim of a Petri dish (14 cm in diameter and 1.5 cm in depth) and the Petri dish was then carefully filled up with DI water. Subsequently, 100 ul UiO-66 suspension (10 mg/ml) was dropped on the glass slide which spread quickly on water surface. After 5 min, 2-3 drops of SDS solution (2wt%) were added to consolidate the UiO-66 film so that a close-packed monolayer was formed. Afterwards, the monolayer was transferred onto the silicon substrate. Finally, the transferred film was dried at 200 ℃ for 20 min. The process was repeated for one, two, or three times and the corresponding thin films were denoted as OTF-SA-1, OTF-SA-2 and OTF-SA-3, respectively.

1.5.4 Direct growth

The composition ratio of the precursor solution is $ZrCl_4$:H_2BDC:H_2O:acetic acid:DMF = 1:1:1:500:1500. In detail, $ZrCl_4$ (0.0933 g) and H_2BDC(0.0655 g) were dissolved in 46.2 mL of DMF. Then, 11.5 mL of acetic acid and 0.0072 mL of water were added to the solution and further mixed at room temperature for 20 minutes. A silicon substrate was then introduced horizontally into a Teflon stainless steel autoclave and the precursor solution was added. The reaction was performed in a 100°C oven for 24 hours. After solvothermal synthesis, the resulting MOFs film was immersed in water and washed several times with water. The resulting film was finally dried at 200 ℃ for 20 min. the according thin films were denoted as OTF-DG.

1.5.5 Layer-by-layer growth

The growth were carried out on thermostatic coating machine (PTL-OV5P, MTI). The functionalized substrate was dipped successively in (1) an ethanolic Zr-precursor (2 mM) solution, (2) absolute ethanol, (3) an ethanolic H_2BDC (2 mM) solution and again (4) absolute ethanol for typically 5 min for each step at 40 ^oC. The studied UiO-66 thin films were prepared in 35 cycles and denoted as OTF-LBL.

1.6 Characterization

Scanning electron microscopy (SEM) images were obtained using a S-4800 electron microscope (Hitachi, Japan). The samples were sputtered with a thin layer of Au prior to imaging. Fourier transform infrared (FTIR) spectra were obtained using a Spectra Two spectrophotometer (PerkinElmer, USA) from 4000 cm^{-1} to 400 cm^{-1} with am attenuated total reflection (ATR) accessory. The X-ray diffraction (XRD) patterns were collected using a Ttr III type X-ray diffractometer (Rigaku, Japan) in θ–θ geometry with a graphite-monochromated CuK_{α} radiation source. The N_2 adsorption-desorption isotherm of the samples at liquid nitrogen temperature (78K) and gas saturation vapor tension range was measured by a BEL Mini sorption instrument. Thermogravimetric analysis (TGA) was completed by a STA6000 thermogravimetric analyzer (Perkin Elmer, Waltham, MA, U.S.) at a scanning rate of 10 ℃ min^{-1} under N_2 atmosphere from 30 to 700 ℃. A fiber spectrometer (USB2000+, Ocean optics, USA) coupled to optical microscope was used to measure the specular reflectance in the 400-900 nm range at the normal incidence. The surface profiles were characterized using a profilometer (Talysurf PGI 1240, Taylor-Hobson, UK) with a diamond stylus. Ellipsometry measurements performed with a XLS-100

ellipsometer (Woollam) at an angle of 75 °, and within a spectral range of 400-900 nm at room temperature.

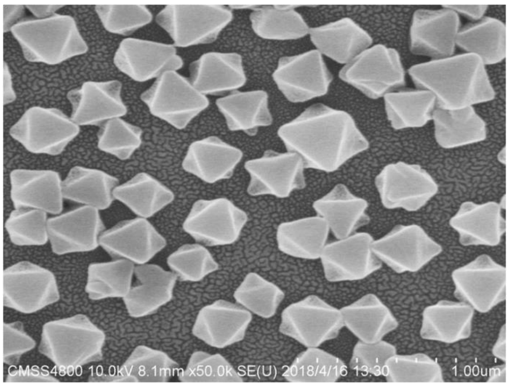

Fig. S1 SEM image of UiO-66 nanocrystals

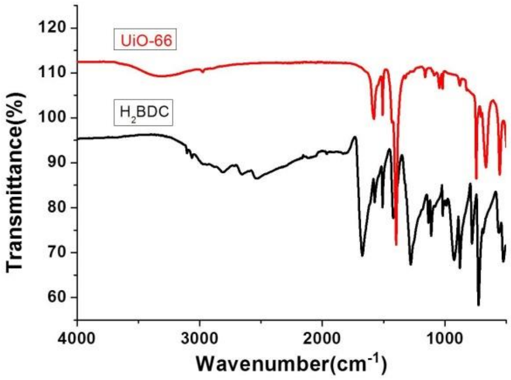

Fig. S2 FTIR ATR spectrum of UiO-66 nanocrystals

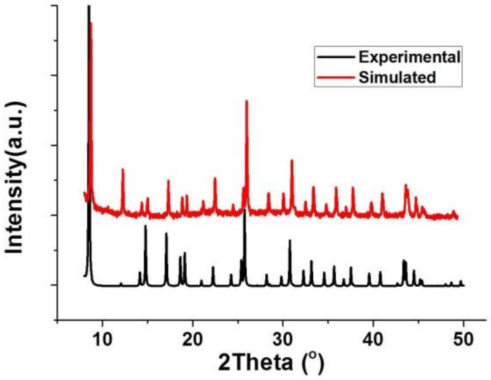

Fig. S3 XRD pattens of UiO-66 nanocrystals

The powder diffraction pattern of UiO-66 nanocrystals was simulated using Mercury 3.10 software and the crystallographic information file from Cambridge Crystallographic Data Centre (CCDC 837796 for UiO-66).

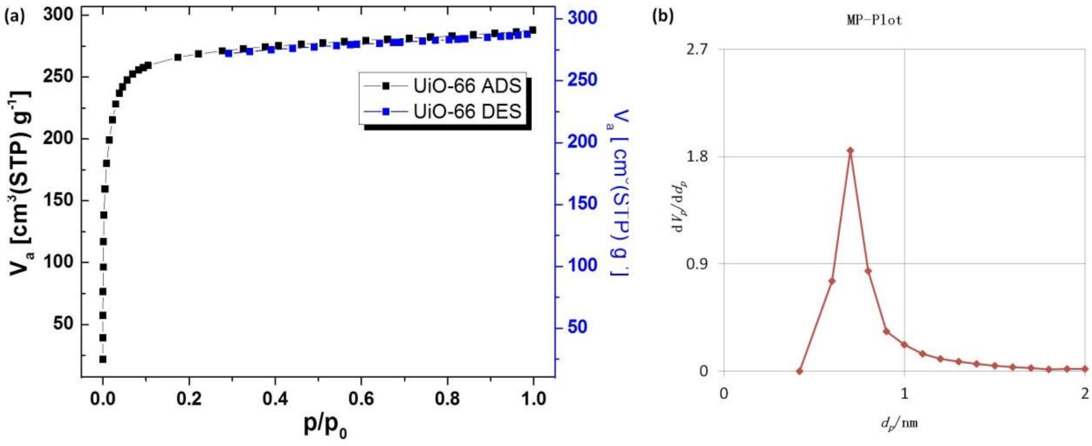

Fig. S4 (a) N_2 adsorption-desorption isotherm and (b) pore size distribution of UiO-66 nanocrystals

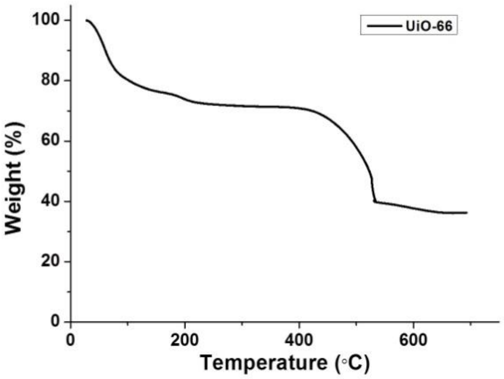

Fig. S5 TGA curve of UiO-66 nanocrystals

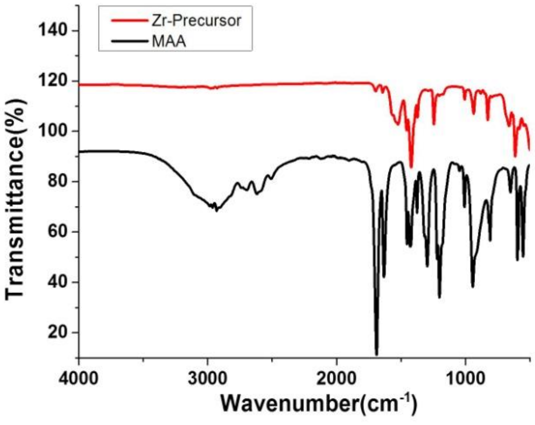

Fig. S6 FTIR ATR spectrum of Zr-precursor

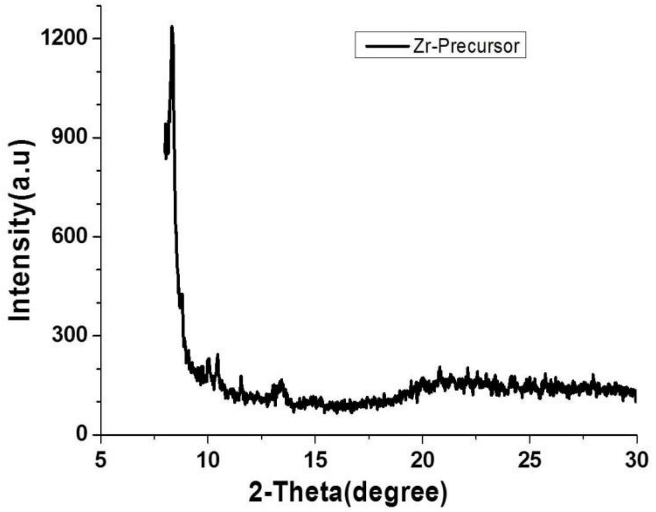

Fig. S7 XRD patten of Zr-precursor

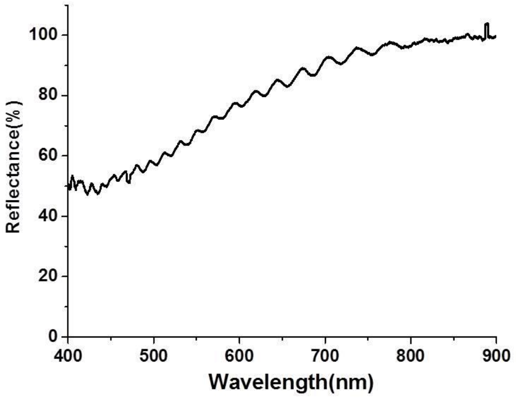

Fig. S8 Specular reflection spectrum of OTF-SP

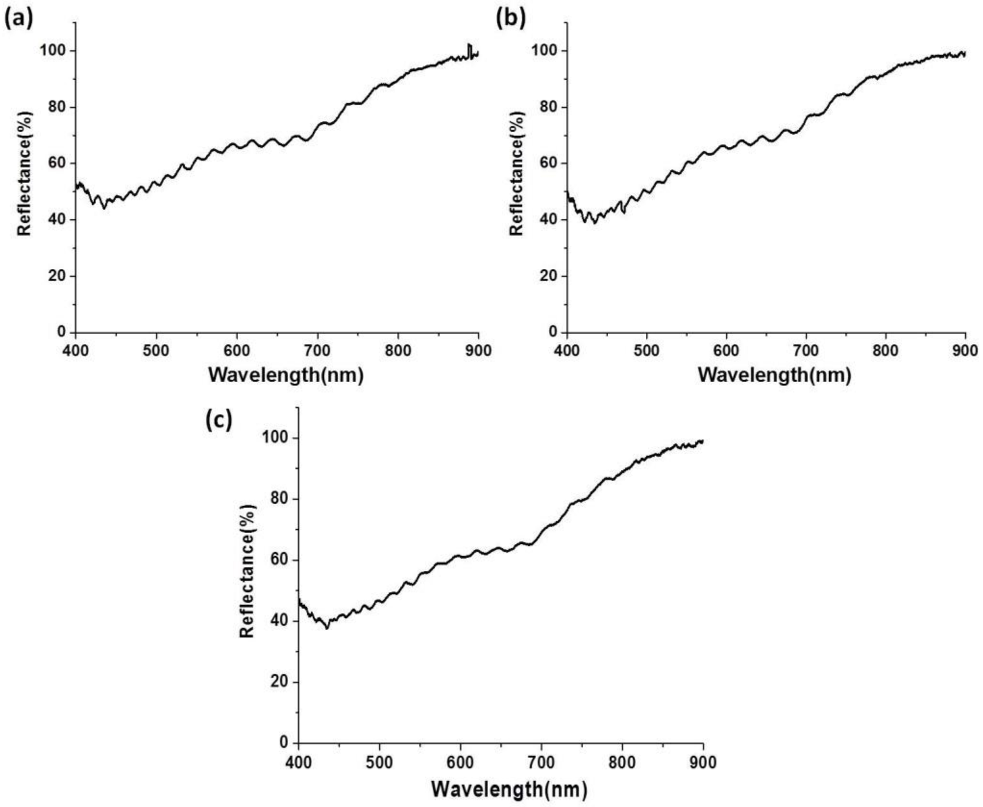

Fig. S9 Specular reflection spectrum of OTF-DP. (a) once, (b) trice, (c) five times.

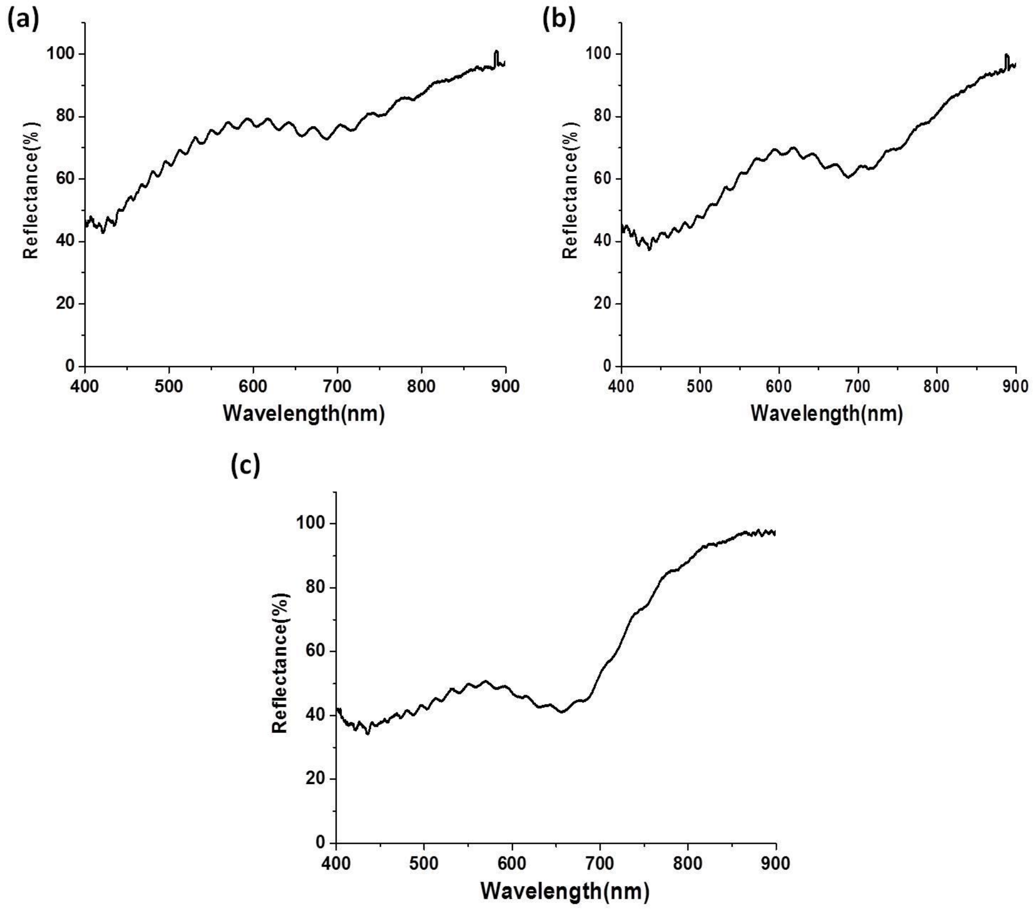

Fig. S10 Specular reflection spectrum of OTF-SA. (a) once, (b) twice, (c) trice.

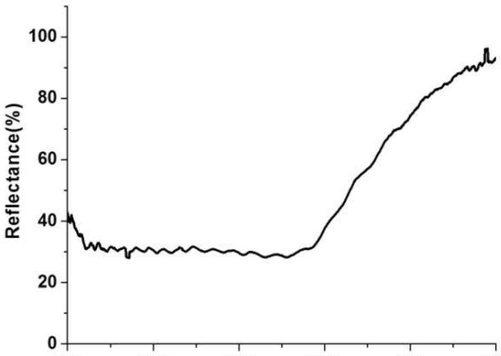

Fig. S11 Specular reflection spectrum of OTF-DG.

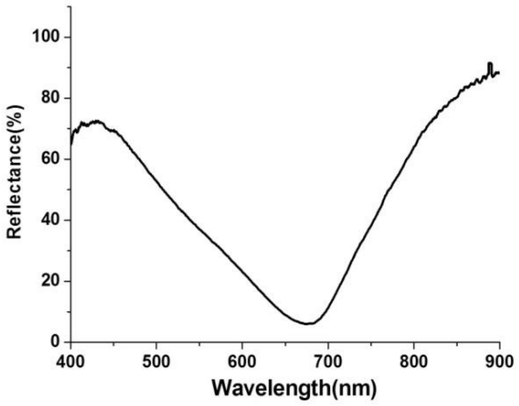

Fig. S12 Specular reflection spectrum of OTF-LBL.

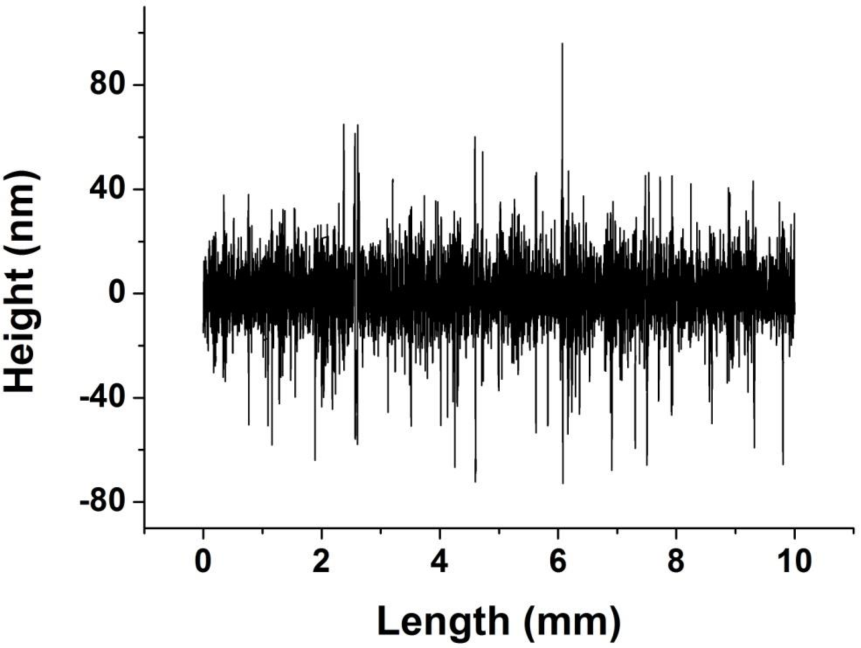

Fig. S13 Surface profile of OTF-SP.

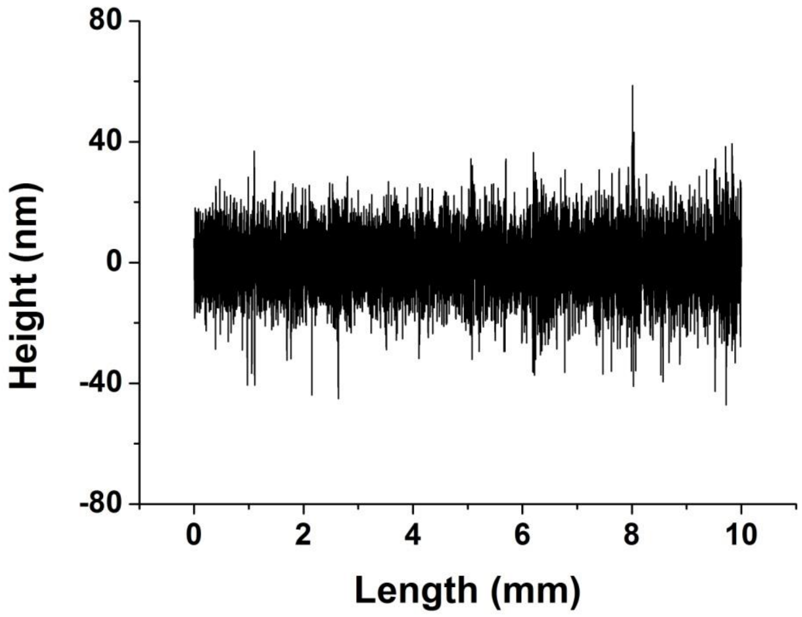

Fig. S14 Surface profile of OTF-DP-1.

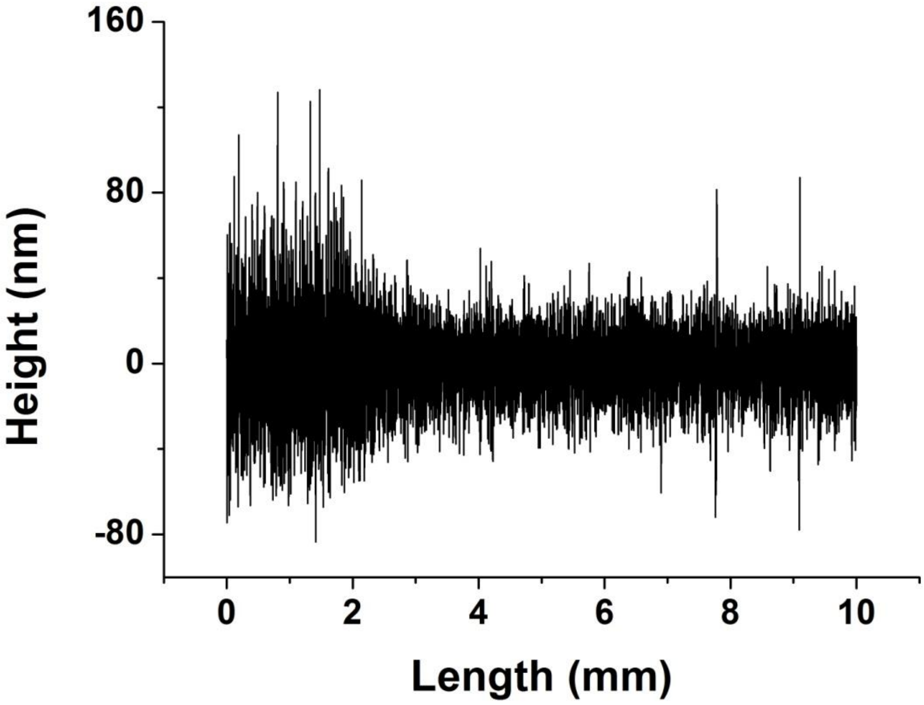

Fig. S15 Surface profile of OTF-DP-3.

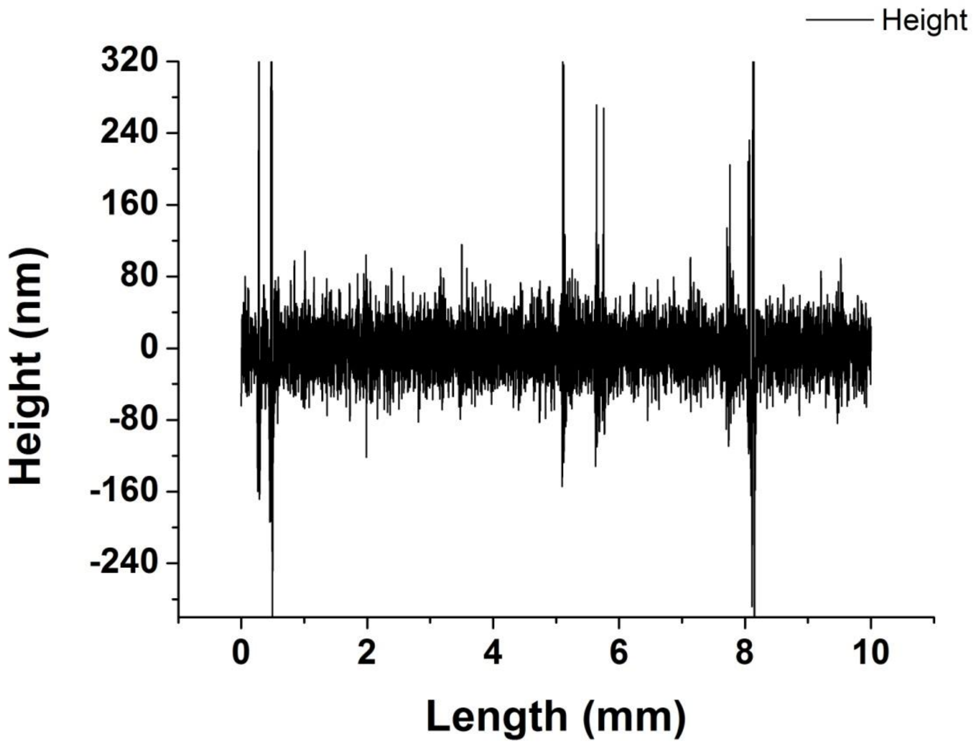

Fig. S16 Surface profile of OTF-DP-5.

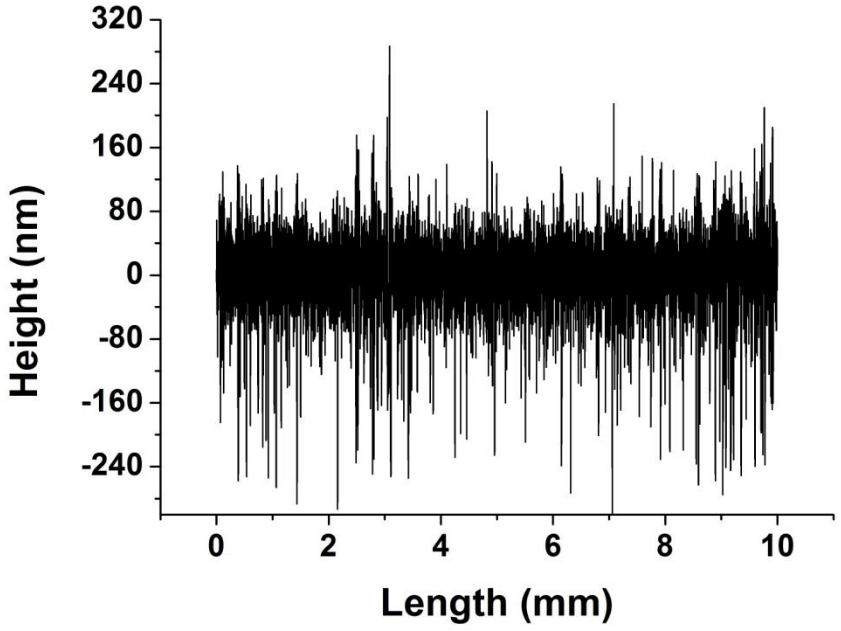

Fig. S17 Surface profile of OTF-SA-1.

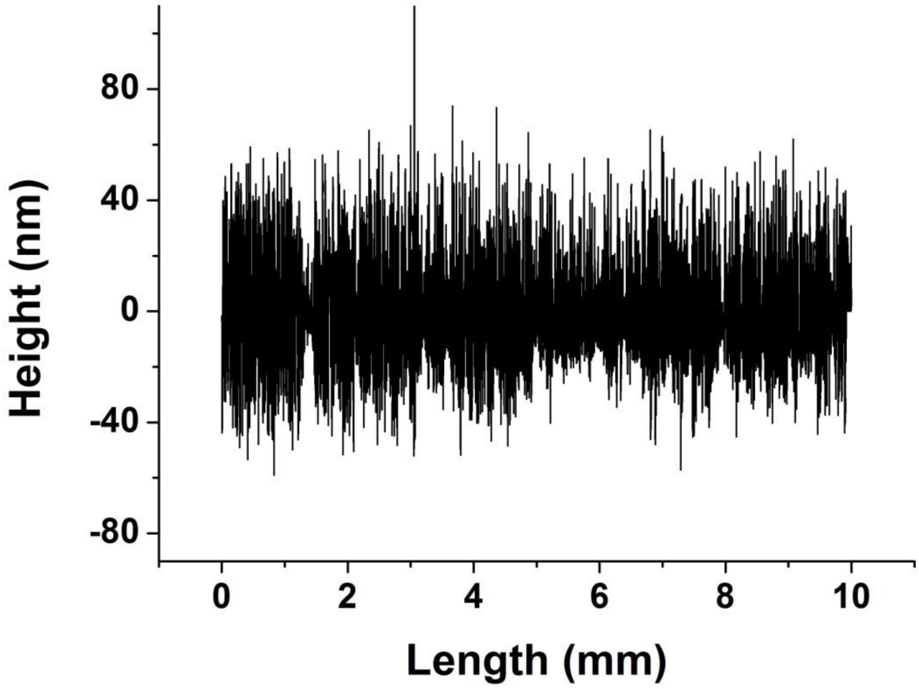

Fig. S18 Surface profile of OTF-SA-2.

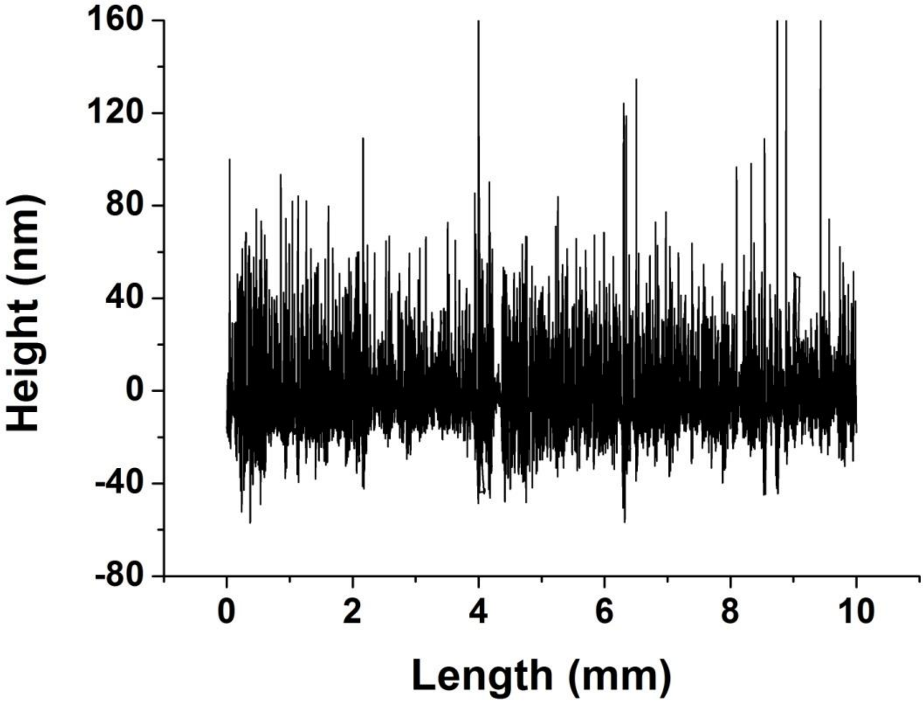

Fig. S19 Surface profile of OTF-SA-3.

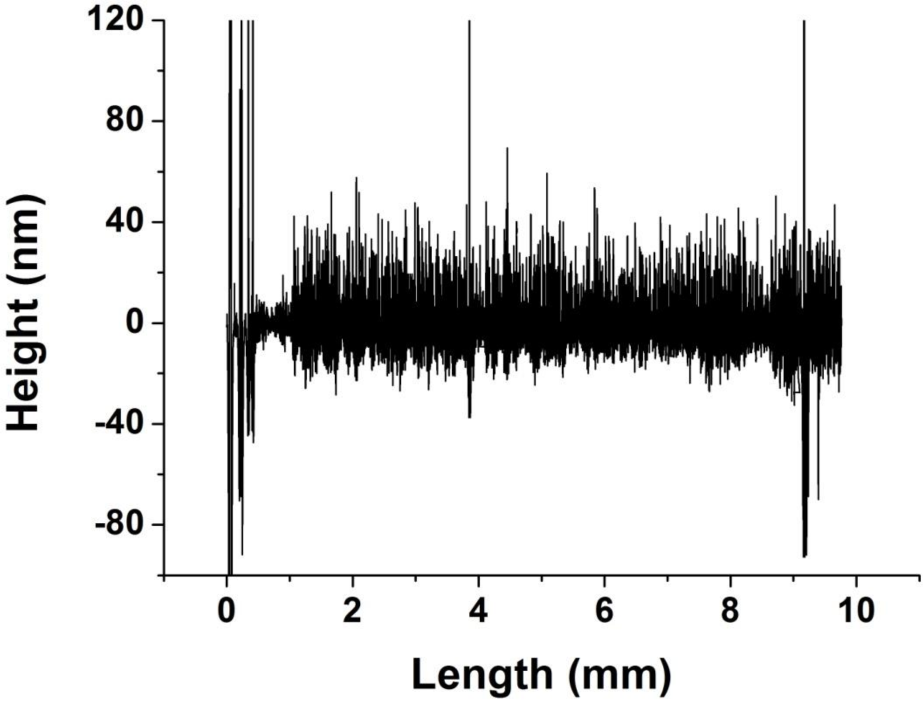

Fig. S20 Surface profile of OTF-DG.

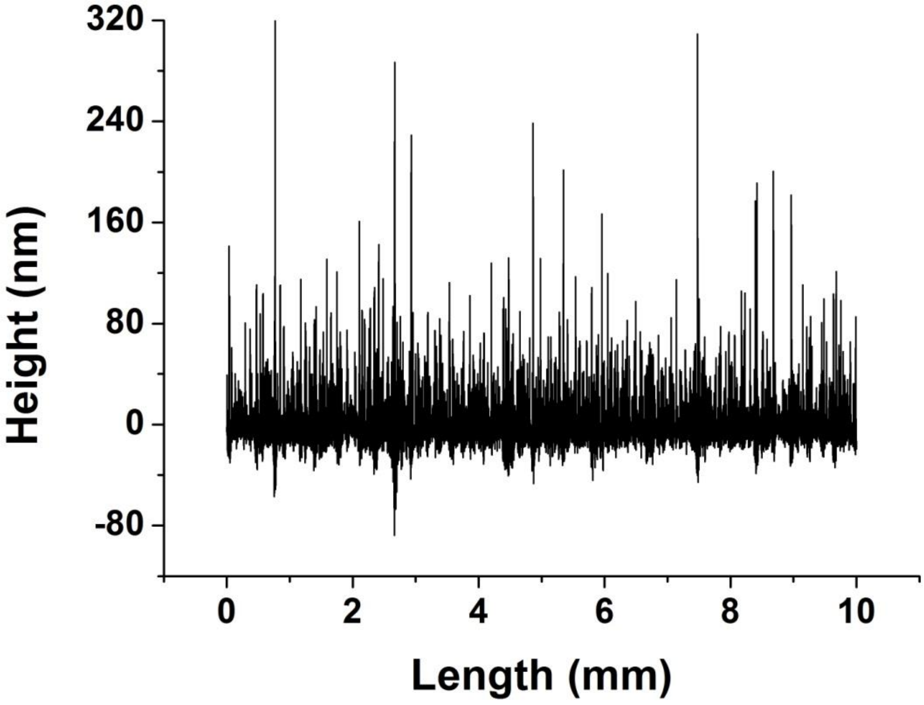

Fig. S21 Surface profile of OTF-LBL.

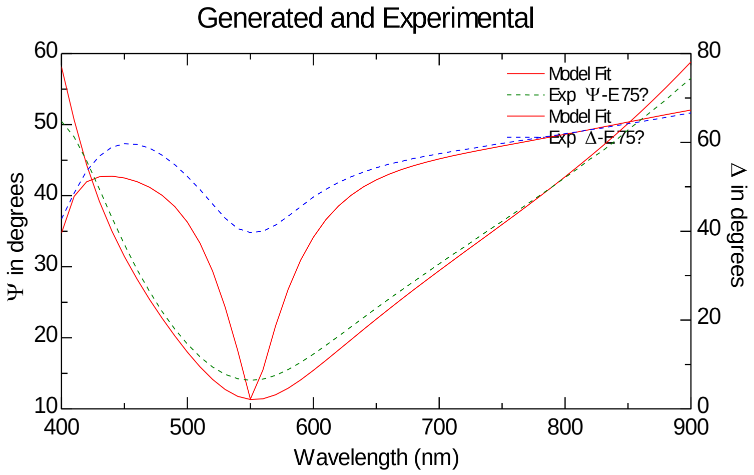

Fig. S22 Generated (solid line) and experimental (dash line) ellipsometry data (Psi and Delta) of OTF-SP.

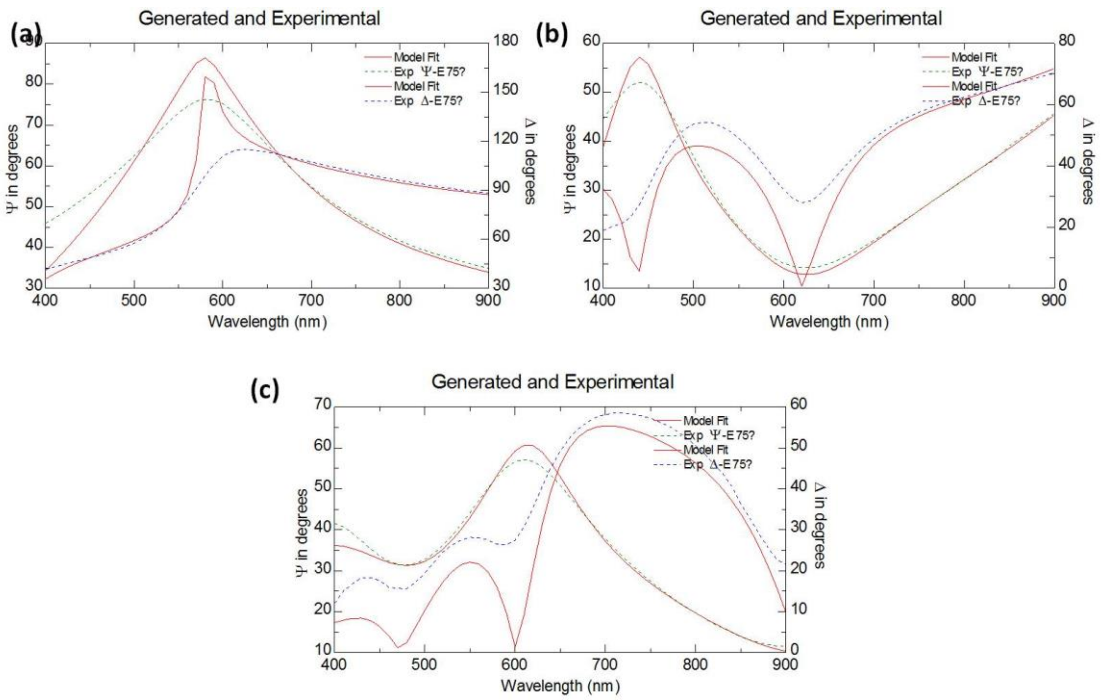

Fig. S23 Generated (solid line) and experimental (dash line) ellipsometry data (Psi and Delta) of OTF-DP. (a) once, (b) trice, (c) five times.

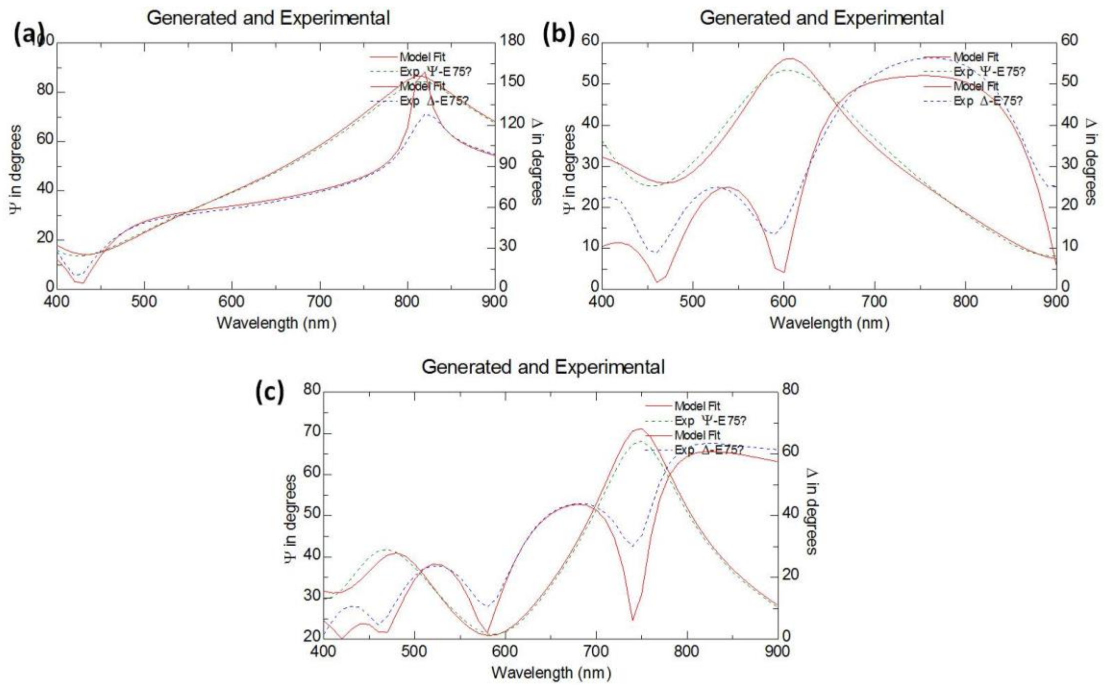

Fig. S24 Generated (solid line) and experimental (dash line) ellipsometry data (Psi and Delta) of OTF-SA. (a) once, (b) twice, (c) trice.

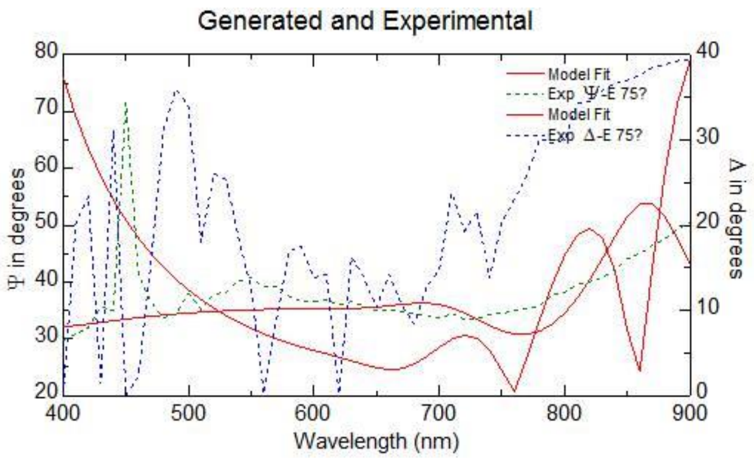

Fig. S25 Generated (solid line) and experimental (dash line) ellipsometry data (Psi and Delta) of OTF-DG.

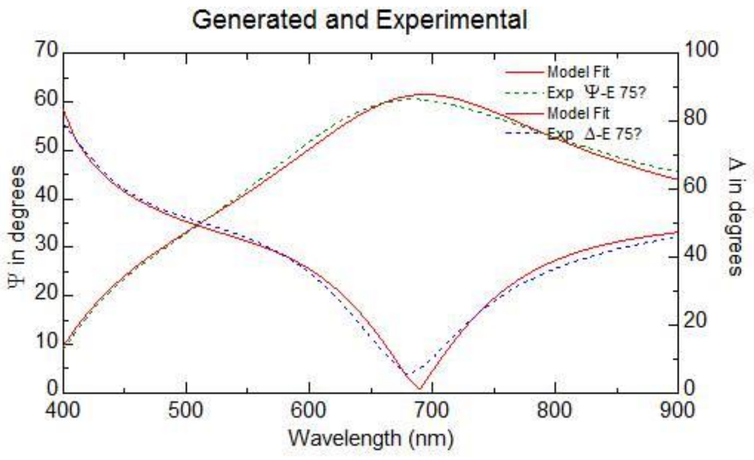

Fig. S26 Generated (solid line) and experimental (dash line) ellipsometry data (Psi and Delta) of OTF-LBL.

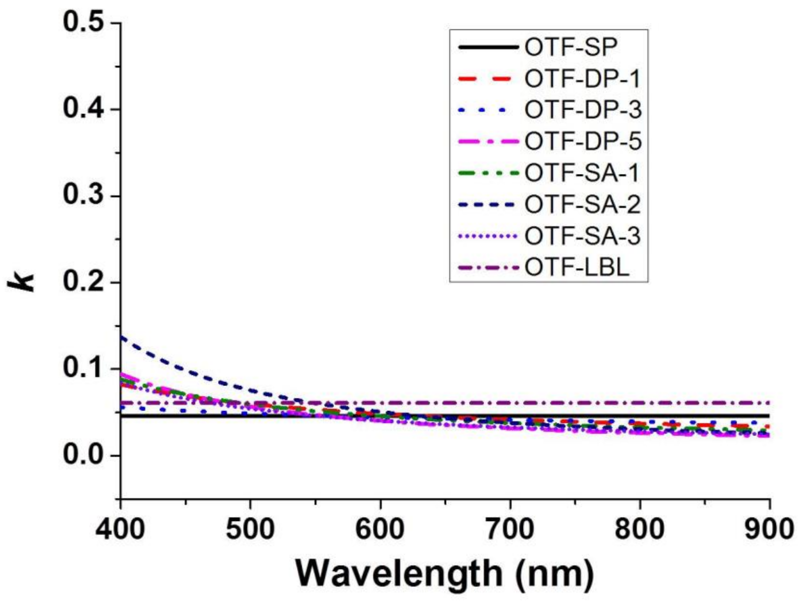

Fig. S27 Efficient extinction coefficient of MOF optical thin films.

Reference

1. K. M. Choi, H. M. Jeong, J. H. Park, Y.-B. Zhang, J. K. Kang and O. M. Yaghi, *ACS nano*, 2014, **8**, 7451-7457.
2. K. S. Park, Z. Ni, A. P. Côté, J. Y. Choi, R. Huang, F. J. Uribe-Romo, H. K. Chae, M. O'Keeffe and O. M. Yaghi, *Proceedings of the National Academy of Sciences*, 2006, **103**, 10186-10191.
3. G. Kickelbick, P. Wiede and U. Schubert, *Inorganica chimica acta*, 1999, **284**, 1-7.
4. K. Užarević, T. C. Wang, S.-Y. Moon, A. M. Fidelli, J. T. Hupp, O. K. Farha and T. Friščić, *Chemical Communications*, 2016, **52**, 2133-2136.
5. C. Cui, Y. Liu, H. Xu, S. Li, W. Zhang, P. Cui and F. Huo, *Small*, 2014, **10**, 3672-3676.
